# On the need for an anticolonial perspective in engineering education and practice

**DOI:** 10.1038/s41467-023-43952-2

**Published:** 2023-12-20

**Authors:** Srinjoy Mitra, Suvobrata Sarkar, Agomoni Ganguli-Mitra

**Affiliations:** 1 School of Engineering, University of Edinburgh, Edinburgh, UK; 2 Department of History, Rabindra Bharati University, Kolkata, India; 3 School of Law, University of Edinburgh, Edinburgh, UK

**Keywords:** Developing world, Engineering

## Abstract

We examine the call for decolonising academic disciplines, and the extent which this applies to engineering. We argue that anticolonial endeavours should systematically recognise colonial legacy in contemporary science and technology, and reframe technological innovation in light of neocolonial extraction and exploitation.

The recent calls^1,2^, in higher education to decolonise the curriculum (and by extension, academic disciplines) are necessary and timely. This stems from the recognition that despite colonised nations having achieved formal independence, the power differentials, economic and political hierarchies between colonisers and those formerly colonised persist in the geopolitical, social, economic, and epistemic realms. Along with what appears to be a renewed and widespread interest in anticolonial approaches and methods however, there is also a well-founded and growing concern that the rush to decolonise serves at best as a ‘metaphor’^3^ for other worthy, but different social/global justice efforts, and that at worst, represents little more than a rebranding and managerial exercise for Global North universities and scholars^2^. Neither effort requires those centres of knowledge production to relinquish their power in the global discourse. As we explore the technology-enabled nature of ongoing neocolonialism and coloniality, we join other voices^4–8^ interrogating power–both its historical acquisition and its continuing implications–to ask what anticolonial work might require from engineering as discipline and practice, within and beyond academia.

## Definitions and positionality

Colonialism is the subjugation and exploitation of one people by another, and often includes the annexation of land and resources (colonisation). Neocolonialism usually refers to ongoing modes of colonialisation, post-formal independence, through for example, the imposition of economic rule and control, exploitation, and cultural imperialism. Coloniality, sometimes used interchangeably with neocolonialism, is often focused on the patterns of power that colonisation has established in the social, epistemic, cultural and political realms, and the legacies that continue to favour and promote Eurocentric domination in these areas. The bodies of academic work associated with anticolonial approaches are vast, and have historically developed as two strands: postcolonial and decolonial approaches. While each is associated with particular histories, geographies and traditions, both terms refer to the scholarship and practices associated with resistance against, and liberation from colonialism (historical and ongoing). In this piece we use the term anticolonial broadly, to refer to any such thought, endeavour or practice, and use specific terms when referring to the relevant scholarship.

Engineering is an uncharacteristic academic discipline in its proximity to the industry, both in terms of research funding and curriculum content^9^. It should be noted that though ‘science’ and ‘mathematics’ appears as separately named disciplines within the commonly used term STEM, the boundaries between applied sciences and applied mathematics on the one hand and, engineering and technology on the other, are often blurred. Our use of the terms science or techno-science refer to all of these applied disciplines. Any call for change therefore cannot be limited to conceptualising engineering as a purely academic endeavour. As academic authors, we hope that our audience will be diverse in terms of its own relationship and proximity to academia and the industry. We also recognise our own positionality and limited scope in offering the following commentary. The first author is a first-generation Indian immigrant, educated in engineering in India and the Global North, trained both in academia and the industry, now working in higher education in the UK; the second author is an Indian historian of science, trained and working in higher education in India; and the third author is a second-generation Indian immigrant and bioethicist exclusively trained in the Global North, working in higher education in the UK. We approach this topic with a recognition of the scope and limitations of our own disciplines and geographies, as well as our different experiences of, and relationships to colonialism and empire.

## Anticolonial endeavours in engineering

A promising starting position, we suggest, is that any anticolonial endeavour in this field requires a recognition and understanding of the historical relationship between technology and empire, and a subsequent investigation into what is said and done in the name of (technological) innovation, growth, development, and progress. As postcolonial theorists have argued, development—and with it, ideas of progress and economic growth—has been, characterised by the ideology that the industrial nations of the Global North are “indubitable models”^10^ for nations of the Global South, that is, for those formerly colonised and exploited by the very nations imposing such a model. Bringing so-called progress to the Global South has involved ushering a new era of industrialisation and urbanisation, and with it social, cultural and structural adjustments. Engineering, through technology and infrastructure, has been a crucial tool in the imposition of such models, in a “post-colonial reaffirmation of (the West’s) perceived superiority”^7^. We recognise that the terms Global North and Global South are themselves problematic, suggesting a false  homogeneity. We have chosen to retain the terms in this case for concision and the broader goal of addressing the global order post 15^th^−20^th^ Century European colonialism and empire, as well as the ongoing power differentials between colonisers and those formerly colonised.

Global North universities have “provided a site through which colonialism–and colonial knowledge in particular–is produced, consecrated and naturalised,”^1^ and it is crucial that we reflect on this legacy within these sites of power, and in conversation with others. We follow Bhambra and colleagues here in suggesting that anticolonial efforts can be pursued without necessarily sacrificing Tuck and Yang’s important political warning on the danger of diluting and misappropriating decolonisation, and that such endeavours require a “way of thinking about the world which takes colonialism, empire and racism as its empirical and discursive objects of study;" and resituate "these phenomena as key shaping forces of the contemporary world”^1^. This of course includes the world of, and shaped by engineering and technology. We argue that various technological endeavours continue to enact certain salient features of colonialism- such as extraction, and the exploitation of land and people- whether or not there is a direct prior relationship of invasion and settler colonialism.

## European techno-science and the creation of engineering as a discipline

At the end of the 15^th^ Century, European economies did not hold a prominent seat in scientific and technological progress^11^^,^^12^. As they  encountered China, the Islamic world and South Asia, Europeans found that they had little to offer these civilisations by way of trade^13^ and techno-scientific knowledge^14^^,^^15^. In addition to several scientific advances that have been documented (and are now understood as important precursors to Eurocentric development^6,16,17^), various non-Western cultures also had advanced technologies in metallurgy, hydrology, textile manufacturing, shipbuilding etc., predating large-scale European arrival (see^18^ for a substantive compilation). It is around this time that European powers invaded the Americas and South Asia, ushering in centuries of brutal exploitation. The development of science and technology progressed alongside the establishment of an empire. The availability of vast newly colonised land and human labour in the form of enslaved people provided the material comfort and prosperity necessary for more people to indulge in life beyond sustenance, allowing for time and resources to be dedicated to the pursuit of certain types of knowledge. In the early days of British science, the trading companies that funded merchants involved in slavery and colonialism played a central role in knowledge production and its funding. An early iteration of the Royal Society was founded by a group of traders who were patrons of the type of technological development that served the needs of colonial projects such as the East India Company. The trading companies supported and employed mathematicians, astronomers, hydrographers, and physicians among others, engaging techno-scientific endeavour in the business of international colonial commerce^17^. As Bala points out, pursuing these ‘mechanical knowledge’ (or technologies) were necessary for Europe to gain prominence in trade and commerce of the existing world^13^. Established scientists (e.g., Robert Boyle, Isaac Newton, and Joseph Banks), acted as directors or major shareholders of institutions similar to the English East India Company^17^^,^^19^. In other words, imperialism and the European applied sciences were inextricably linked from their early days.

Several aspects of the Scientific Revolution take on a different shade when observed through an anticolonial lens. As Harding and others have pointed out, a focus on the causal relation between the development of modern sciences in Europe and the “voyages of discovery” reframes what has traditionally been conceptualised as ‘the Scientific Revolution’, as well as the inherent “triumphalism” of Western science/technology^20^. For example, Newton’s access to data on tides, pendulums, and comets heavily depended on the existence of the Atlantic slave trade. Along with French astronomers, such data were often recorded by locals whose names were rarely documented^21^. Similarly, the rise of natural history research in Western Europe (from Sloane, to Humboldt and Darwin) is deeply connected to imperial trade. The importance—ordinarily eclipsed within the narrative of progress and modernity—of such encounters, and the contribution of local interlocutors and local knowledge in developing modern science and technology is only now being systematically examined^17,22^.

The steam engine, commercialised by James Watt who expressed gratitude to enslavers in the Caribbean for their financial support^11^, was a key feature of the industrial revolution. This new technology was put to use for efficient sugar refinement in the plantations in order to meet the needs of European elites and the growing working class^23^. Technological innovation in steamships was central to the ‘triangular trade’ of enslaved people, goods and precious metals^24^. Further development in steam power, for example, by Carnot and Kelvin, was used in mining colonial resources as well as in the ‘Scramble for Africa’^25^. Prominent figures of 19^th^ Century British science, such as Faraday, Kelvin, Maxwell, were not theorising over abstract electrodynamics but were directly connected to the technological development of long-distance electric telegraph^26^. These underwater cables connected the Empire, and were of key military significance in crushing revolts in the colonies, such as the 1857 Sepoy Rebellion in India^27^. Kelvin and others reaped immense financial benefits from being consultants to these British technology companies and their imperial and commercial success^28^. One of the indispensable ingredients in this particular development was the ‘discovery’ and extensive use of gutta percha (a natural rubber, well-known to the local community and introduced to a British officer by an unnamed Malay man) as the underwater cable insulator^29^. Painstakingly collected by local labour, so immense was Empire’s demand for the rubber that by early 1900, the wild gutta percha tree was almost driven to extinction and led to a local ecological disaster^30^.

The rise and expansion of industrial Europe, particularly in the period between 1850 and 1914, was characterised by an “explosive increase of European population and its movement overseas, and the rise of modern capitalist economy and its evolution into industrialism”^31^, increasing the dependency of colonised nations on the technologies of colonisers. Beyond the direct examples of military technology developed to dominate the colonies, empire-makers saw opportunities for technological experimentation and development that were not feasible in Europe. India for example, served as a ‘laboratory’ for the new railway industry, a large experimental ground where mechanical engineers tested their skills and adapted their practices. Other examples include the electrification of large urban neighbourhoods in Calcutta, the second largest city of the Empire, as well as test beds of shipbuilding^32^. Working within the empire resulted career opportunities for engineers, providing “professional expertise to many individuals who in turn made significant contributions to European technology—in such fields as mining, bridge-building, water-management and medicine”^33^. In colonial India, the Thomason Civil Engineering College was established in Roorkee (1847) following the Ganga irrigation project^32^. Its success in creating  local expertise  for public projects led to the establishment  of several institutions. In fact, these Indian engineering institutions provided models that were replicated in formal engineering education in England^34^. Throughout the eighteenth and nineteenth centuries, a growing number of graduates from European universities “were absorbed into the ever-expanding overseas services of trading groups to occupy senior technical positions. (…) Some of these were to become prominent men of science.”^17^.

Recognising modern science and technology as a phenomenon deeply entangled with the violent ideology, practices, and objectives of empire and colonisation provides a different context to how we consider the ethics and ethos of engineering, and indeed to the meaning of techno-scientific progress and innovation. Historical evidence suggests that colonisation, and large-scale engineering experiments within colonies, not only created engineers, but much of the discipline of (civil) engineering as we recognise it today. It should be noted that other than artisanship, engineering as a discipline (in Europe) was traditionally connected to militaristic needs, and ‘civil-engineering’ was a term first coined in 18^th^ century to designate  all non-military engineering practices^35^. Of course, the various legacies of colonialism are not restricted to engineering and technology. As Seth argues, “the history of almost all modern science, it has become clear, must be understood as ‘science in a colonial context’”^36^. Unfortunately, both engineering education and practice—and indeed much STEM education and practice—are currently devoid of such historical context. Engineering and technology were important tools of colonisation, but colonisation also provided resources that made technology-enabled power in the Global North possible. The construction of European technical superiority was as much material as it was ideological, founded on the moral imperative of a God-bestowed right and responsibility to govern the natural world—including racialised and indigenous people—that required taming and civilising. Along with the development of highly visible industrial technologies, the ideology of technological (hence civilisational) superiority grew, reinforcing the idea of progress as belonging to the colonisers. Machines became the universal “measure of men”^37^.

## Technological dominance as ongoing coloniality

As Tuck and Yang have argued, a central characteristic of colonialism lies in the “the expropriation of fragments of Indigenous worlds, animals, plants and human beings, extracting them in order to transport them to—and build the wealth, the privilege, or feed the appetites of—the colonisers, who get marked as the first world”, while those who bore the brunt of colonialism are left to “un-underdevelop” themselves with various forms of interventions (political, economic, cultural and technological) from the Global North. In many disciplines, people, samples and data from the Global South have been systematically treated as resources that researchers in the North could exploit to their own benefit, without meaningful collaboration or acknowledgement^3,38^. While 21^st^ century has seen some progress in academic collaboration with Global South partners, the coloniality of such ‘helicopter/parachute research’ is still largely prevalent^39,40^. As pointed out by several scholars^7,41^, the well-intentioned £1.5 billion Global Challenge Research Fund (GCRF) launched by the UK government in 2016 assumed a similar neocolonial approach, and has been taken up with enthusiasm by the engineering and technology community.

Where does this leave us? An initial step in thinking about anticolonial perspectives in engineering we suggest, lies in the systematic recognition by the discipline—within and beyond academia—of this history and colonial legacy in contemporary science and technology. Not only as a part of undergraduate curriculum—and in response to calls to decolonise curricula in the Global North—but also as part of ongoing training for technologists and engineering researchers, within and beyond academia. A second, and potentially urgent imperative however, lies in the recognition of how the historically established global order, one that has been enabled by, and contributed to, engineering progress, continues to engage with, and reinforce colonial structures and processes. Once we view the current global order as one built on power acquired through colonisation, we can begin to see that the coloniality sustained by technological progress (and associated economic power) is alive and well.

The incessant demand for economic growth is both a cause, and an outcome of technological innovation, primarily driven by capitalist and technocratic ideologies emerging in the Global North^42,43^. But this comes at an enormous cost, exemplified by the present climate crisis and ecological breakdown, systematically and disproportionally affecting those marginalised by hierarchies of power. Instead of focusing on behavioural and institutional changes to reduce energy demand, the promise of novel green-tech solutions to the climate crisis continues to rely on colonial ideologies of extractivism and waste-disposal to the Global South. No existing technology (e.g., various clean electricity and carbon capture options that the IPCC, Inter-Governmental Panel on Climate Change, heavily depends on) has the capacity to meet the COP26 goals^44^, and more than 80% of major US-based CCS (Carbon Capture and Storage) projects have failed to demonstrate commercial viability^45^. Were ‘green-technologies’ to be scaled globally, deploying such interventions would require an unprecedented increase in material footprint^46,47^. Currently, such resources are primarily obtained from the Global South: Lithium (for batteries) from Bolivia, conflict minerals (for various manufacturing) from DR Congo, cheap water or energy from South Asia (where most semiconductor manufacturing takes place)^48^ and rare-earth materials (for windmills to solar panels) from Inner Mongolia^49^. In continuing our “imperial mode of living”^43^, the North already has a footprint many times higher than the sustainable limit, and such material extraction will continue to cause irreversible damage to the vulnerable local ecologies of the South^50^. Unsurprisingly, e-waste and factory pollutants already cause disproportionate harm to these very populations. While the African continent is home to some of the world’s largest solar power plants, and sells power to the European grid, several African regions continue to face immense challenges in accessing basic electricity^51^. Similarly, the global network of cables that forms the backbone of the internet crosses many geographical boundaries, but not everyone benefits from its existence. For example, Eritrea allows 12 cables to pass through the nation, without a single access point within its national boundaries. Similarly, several African and South American countries share a limited number of marine cables without backup in case of breakage^29^. The technology driven neocolonial future looks even bleaker if we consider calls for planetary-scale geoengineering projects to mitigate climate change, primarily driven by Global North actors^52^. This deliberate, large-scale intervention in the Earth’s climate system might have several unknown consequences whose negative effects are likely to be borne by people who contributed the least to the climate crisis^53^.

The maintenance of a capitalist-consumerist power hierarchy requires the continued exploitation and extraction of lands, resources and knowledge, while also engaging in continued denial of the very humanity of those being exploited^38^. There is an unquestioned imperative to innovate and grow in the engineering sector which needs to be countered by an ethical imperative to question this growth, one that  is motivated by the understanding of historical and ongoing coloniality. When we think about a moral prescription in ethics, we consider not only the question: ‘what is the right thing to do?’ but also ‘what do we have an obligation to do’ or ‘to refrain from doing’? We would argue that engaging in a different type of thinking, training and practice is an ethical obligation for engineering researchers and technologists from the Global North, who continue to drive—and benefit from—neocolonialism and various forms of coloniality.

## Conclusion

In thinking about the ethics of engineering, it is common to come across the term ‘responsible innovation’. The aim is to innovate in a manner that reduces harm and increases benefit, but to innovate, nonetheless. What remains largely unquestioned within such a framing is a relentless drive towards techno-solutionism (the idea that most problems have a technological solution). Underlying this is an ideology fuelled by techno-optimism (the idea that technology is more beneficial than harmful, or that technology is crucial to solving the greatest problems facing humankind). As argued by various postcolonial and decolonial scholars^10,20,38,54^, simply proceeding with an idea of ‘doing good’, devoid of the geopolitical and historical understanding of interventions, and deployment of technological products and infrastructure, continue to recreate and embed the legacies of colonialism.

The ethical obligation to counter coloniality, must begin with considering how technological innovation can be sustainable and defensible in light of the ongoing extraction and exploitation of lands and people. A promising point of departure will be in engaging with the existing and growing interdisciplinary postcolonial and decolonial scholarship, and in dialogue with those who are working in similar directions^8^.  We are adding our voices to those critiquing the so-called neutrality and objectivity of engineering^5^ and its “ideology of depoliticization”^55^. Emerging anticolonial discussions in global health^56,57^, a field that is beginning to reckon with its own colonial past and legacies, may provide an important site of dialogue. Similarly, anticolonial work in engineering can build on, interrogate, and extend the broader work on ethics and justice^8,58–60^, given that social justice is “integral to decolonisation”^2^. Importantly, while academics and institutions from the Global North have a role to play in this work, we must refrain from enacting further material harm and epistemic erasure by offering models and solutions developed predominantly in the North, or for that matter, by leading on anticolonial efforts on a global scale.

Within the Global North, and especially within Global North Universities, an anticolonial approach to engineering, must consider where and how it might contribute to reparation, restitution, as well as to power and material redistribution. Given academic engineering’s role in establishing norms, practices, and methods, we must question the colonial outlook of our Eurocentric curriculum and practices. Necessarily, such approaches will be heterogenous, perhaps as diverse as actors, and their specific positions of (geographical, historical, or disciplinary) privilege. As Gopal argues, anticolonialism can give rise to “different kinds of resistance” to colonialism. This piece is one such call to resistance.

## References

[1] Bhambra, G. K., Gebrial, D. & Nişancıoğlu, K. Introduction: Decolonising the Unversity?, in *Decolonising the University*, Pluto Books (2018).

[2] Gopal P (2021). On Decolonisation and the University. Textual Pract..

[3] Tuck E, Yang KW (2012). Decolonization is not a metaphor York at New Paltz. Decolonization Indig. Educ. Soc..

[4] Muller, M. Decolonising engineering in South Africa – Experience to date and some emerging challenges. *South Afr. J. Sci.* **114** (2018).

[5] Lord, S. M., Mejia, J. A., Hoople, G. D. & Chen, D. A. Starting a dialogue on decolonization in engineering education. in *2019 IEEE Frontiers in Education Conference (FIE)*, Covington, KY, USA: IEEE, 2019, pp. 1–3. 10.1109/FIE43999.2019.9028477.

[6] Delbourgo J (2019). The knowing world: a new global history of science. Hist. Sci..

[7] Eichhorn SJ (2020). How the West was won: a deconstruction of politicised colonial engineering. Polit. Q..

[8] B. Momo, G. D. Hoople, D. A. Chen, J. A. Mejia, and S. M. Lord, Broadening the Engineering Canon: How Culturally Responsive Pedagogies Can Help Educate the Engineers of the Future, *Murmurations Emergence Equity Educ*., vol. 2.

[9] Mitra, S. & Raskin, J.-P. The paradox of industrial involvement in engineering higher education. in *IEEE Frontiers for Engineering Education*, IEEE, (2023).

[10] Escobar, A. Encountering Development – The Making and Unmaking of the Third World: 1, With a New preface by the author edition. Princeton, N.J: Princeton University Press (2011).

[11] Andrews, K. *The New Age of Empire*. Allen Lane (2021).

[12] Headrick, D. *Power Over Peoples*. Princeton University Press (2010).

[13] Bala, A. *The Needham Question and Southeast Asia: Comparative and Connective Perspectives*. in The Bright Dark Ages. BRILL (2016).

[14] Goldstone, J. *Why Europe? The Rise of the West in World History 1500–1850*, 1st edition. Boston: McGraw-Hill Education (2008).

[15] Poskett, J. *Horizons: A Global History of Science*. London, UK: Viking (2022).

[16] Bala, A. *The Dialogue of Civilizations in the Birth of Modern Science*. New York: Palgrave Macmillan US, 2006. 10.1057/9780230601215.

[17] Kapil Raj, *Relocating Modern Science Circulation and the Construction of Knowledge in South Asia and Europe, 1650–1900*. Palgrave Macmillan (2007).

[18] Selin, H. Ed., *Encyclopaedia of the History of Science, Technology and Medicine in Non-Western Cultures*. Springer (2016).

[19] Odlyzko A (2018). Newton’s financial misadventures in the South Sea Bubble. Notes Rec. R. Soc. J. Hist. Sci..

[20] Harding S (2009). Postcolonial and feminist philosophies of science and technology: convergences and dissonances. Postcolonial Stud..

[21] Dew, N. Circulating measurements around the French Atlantic, in *Science and Empire in the Atlantic World*, Dew, N. & Delbourgo, J. (Eds.), Routledge (2008).

[22] Delbourgo, J. *Collecting the World: Hans Sloane and the Origins of the British Museum*. Harvard University Press (2019).

[23] Williams, E. *Capitalism and Slavery*. The University of North Carolina Press (1944).

[24] Smith, C. & Wise, M. N. *Energy and Empire: A Biographical Study of Lord Kelvin*. Cambridge University Press (1989).

[25] Headrick, D. *Tools of the Empire*. Oxford University Press, 1981.

[26] Schaffer S (2011). The laird of physics. Nature.

[27] Headrick D, Double-Edged A (2010). Sword: Communications and Imperial Control in British India,. Hist. Soc. Res..

[28] Hunt, B. J. *Imperial Science*. Cambridge University Press (2021).

[29] Thorat D (2019). Colonial topographies of internet infrastructure: the sedimented and linked networks of the telegraph and submarine fiber optic internet. South Asian Rev..

[30] Tully J (2009). A victorian ecological disaster: imperialism, the telegraph, and gutta-percha. J. World Hist..

[31] C. B. Goh, *Technology and entrepôt colonialism in Singapore, 1819*–*1940*. Institute of South Asian Studies, 2013.

[32] R. Macleod and D. Kumar, Eds., *Technology and the Raj: Western Technology and Technical Transfers to India*. Sage, 1995.

[33] Arnold D (2005). Europe, technology, and colonialism in the 20th century. Hist. Technol..

[34] R. Dionne and R. Macleod, Science and Policy in British India, 1858–1914: Perspectives on a Persisting Belief. in *Proceedings of the Sixth European Conference on Modern South Asian Studies*. CNRS, 1979.

[35] A. W. Skempton, *A Biographical Dictionary of Civil Engineers Volume 1, 1500*–*1830*. ICE Publishing, 2002.

[36] Seth S (2009). Putting knowledge in its place: science, colonialism, and the postcolonial. Postcolonial Stud..

[37] Adas, M. *Machines as the Measure of Men*. Cornell University Press (2014).

[38] Tuhiwai Smith, L. *Decolonizing Methodologies*. Bloomsbury (2016).

[39] Editorial, Nature addresses helicopter research and ethics dumping, *Nature*, vol. 606, 7912, pp. 7–7, May 2022, 10.1038/d41586-022-01423-6. 10.1038/d41586-022-01423-6 35637322

[40] Odeny B, Bosurgi R (2022). Time to end parachute science. PLoS Med..

[41] Noxolo P (2017). Decolonial theory in a time of the re-colonisation of UK research. Trans. Inst. Br. Geogr..

[42] Hickel, J. *Less is More: How Degrowth Will Save the World*. Windmill Books (2021).

[43] Schmelzer, M., Vetter, A. & Vansintjan, A. *The Future is Degrowth: A Guide to a World Beyond Capitalism*. New York: Verso (2022).

[44] Allwood, J. Technology will not solve the problem of climate change, *Financial Times*, Nov. 16, 2021. [Online]. Available: https://www.ft.com/content/207a8762-e00c-4926-addd-38a487a0995f.

[45] A. K. Bhowmik and N. Grant, Relying on carbon capture to solve the climate crisis risks pushing our problems into the next generation’s path, *The Conversation*, 2022. http://theconversation.com/relying-on-carbon-capture-to-solve-the-climate-crisis-risks-pushing-our-problems-into-the-next-generations-path-175269 (accessed Sep. 23, 2022).

[46] Södersten C-J, Wood R, Wiedmann T (2020). The capital load of global material footprints. Resour. Conserv. Recycl..

[47] Hickel J, Slamersak A (2022). Existing climate mitigation scenarios perpetuate colonial inequalities. Lancet Planet. Health.

[48] Belton, P. The computer chip industry has a dirty climate secret, *The Guardian*, 2021. Accessed: Mar. 26, 2023. [Online]. Available: https://www.theguardian.com/environment/2021/sep/18/semiconductor-silicon-chips-carbon-footprint-climate.

[49] Pitron, G. *The Rare Metals War: the dark side of clean energy and digital technologies*. London, UK Brunswick, Australia Minneapolis, US: Scribe UK, 2021.

[50] Hickel J, Kallis G (2020). Is green growth possible?. N. Polit. Econ..

[51] Táíwò, O. O. The Green New Deal and the Danger of Climate Colonialism, *Slate*, Mar, 2019. Accessed: Mar. 05, 2023. [Online]. Available: https://slate.com/technology/2019/03/green-new-deal-climate-colonialism-energy-land.html.

[52] Stephens JC, Surprise K (2020). The hidden injustices of advancing solar geoengineering research. Glob. Sustain..

[53] Carvalho A, Riquito M (2022). ‘It’s just a Band-Aid!’: public engagement with geoengineering and the politics of the climate crisis. Public Underst. Sci..

[54] Mohanty CT (1988). Under western eyes: feminist scholarship and colonial discourses. Fem. Rev..

[55] Cech EA, Sherick HM (2015). Depoliticization and the structure of engineering education. Int. Perspect. Eng. Educ. Eng. Educ. Pract. Context.

[56] Abimbola S, Pai M (2020). Will global health survive its decolonisation?. Lancet.

[57] Bhakuni H, Abimbola S (2021). Epistemic injustice in academic global health. Lancet Glob. Health.

[58] Riley, D. Engineering and Social Justice. in *Synthesis Lectures on Engineers, Technology, & Society*. Springer International Publishing, 2008.

[59] Lucena, J., Ed., *Engineering Education for Social Justice: Critical Explorations and Opportunities*, vol. 10. in Philosophy of Engineering and Technology, vol. 10. Dordrecht: Springer Netherlands, 2013. Accessed: Mar. 22, 2023. [Online]. Available: https://link.springer.com/10.1007/978-94-007-6350-0

[60] Benjamin, R. *Race After Technology*. Polity, 2019.

